# Wrist Circumference-Dependent Upper Limit of Normal for the Cross-Sectional Area Is Superior Over a Fixed Cut-Off Value in Confirming the Clinical Diagnosis of Carpal Tunnel Syndrome

**DOI:** 10.3389/fneur.2021.625565

**Published:** 2021-02-05

**Authors:** Tom B. G. Olde Dubbelink, Floriaan G. C. M. De Kleermaeker, Roy Beekman, Juerd Wijntjes, Ronald H. M. A. Bartels, Jan Meulstee, Wim I. M. Verhagen

**Affiliations:** ^1^Department of Neurology, Canisius-Wilhelmina Hospital, Nijmegen, Netherlands; ^2^Department of Neurology, VieCuri Medical Centre, Venlo, Netherlands; ^3^Department of Neurology, Zuyderland Medical Centre, Heerlen, Netherlands; ^4^Department of Neurology, Radboud University Medical Centre, Nijmegen, Netherlands; ^5^Department of Neurosurgery, Radboud University Medical Centre, Nijmegen, Netherlands

**Keywords:** carpal tunnel syndrome, ultrasonography, cross-sectional area (CSA), median nerve, cut-off

## Abstract

**Introduction:** In confirming the clinical diagnosis of carpal tunnel syndrome (CTS), ultrasonography (US) is the recommended first diagnostic test in The Netherlands. One of the most important parameters for an abnormal US result is an increase of the CSA of the median nerve at the carpal tunnel inlet. An earlier study showed that a wrist-circumference dependent cut-off for the upper limit of normal of this CSA might be superior to a fixed cut-off of 11 mm^2^. In this study we compared three ultrasonography (US) parameters in three large Dutch hospitals.

**Methods:** Patients with a clinical suspicion of CTS and with reasonable exclusion of other causes of their symptoms were prospectively included. A total number of 175 patients were analysed. The primary goal was to compare the number of wrists with an abnormal US result while using a fixed cut-off of 11 mm^2^ (FC), a wrist circumference-dependent cut-off (y = 0.88 ^*^ x−4, where y = ULN and x = wrist circumference in centimetres; abbreviated as WDC), and an intraneural flow related cut-off (IFC).

**Results:** The WDC considered more US examinations to be abnormal (55.4%) than the FC (50.3%) did, as well as the IFC (46.9%), with a statistically significant difference of *p* = 0.035 and *p* = 0.001, respectively. The WDC detected 12 abnormal median nerves while the FC did not, and 18 while the IFC did not. The wrist circumference of the patients of these subgroups turned out to be significantly smaller (*p* < 0.001) when compared with the rest of the group.

**Conclusion:** According to these study results, the wrist-circumference dependent cut-off value for the CSA of the median nerve at the wrist appears to have a higher sensitivity than either a fixed cut-off value of 11 mm^2^ or cut-off values based on intraneural flow, and may add most value in patients with a smaller wrist circumference.

## Introduction

Carpal tunnel syndrome (CTS) is the most common peripheral mononeuropathy with a prevalence ranging from 1 to 6% in the general population (1, 2). CTS is caused by compression of the median nerve as it travels through the carpal tunnel, and can be diagnosed clinically. The symptoms classically include pain and paraesthesia in the territory of the median nerve, increasing during the night, and provocation by flexing or extending the wrist (3). Case history evaluation is the most important part of consultation for the clinical diagnosis of CTS (4). Nevertheless, in The Netherlands, most surgeons require a confirmation by an electrodiagnostic or ultrasound test (5).

As of 2017, ultrasonography (US) is the recommended first diagnostic test in The Netherlands because it is easily accessible and painless. Moreover, US and nerve conduction studies (NCS) have a similar sensitivity and specificity, according to the Dutch CTS guideline (6). In literature, several ultrasonography parameters for confirming the diagnosis CTS are suggested, the most important being (7, 8):

- An increase in the cross-sectional area (CSA) of the median nerve at wrist level;
- Flattening ratio of the median nerve at the hamate level;
- Swelling ratio; increase in the CSA at the wrist level compared to the CSA at distal radius level (9);
- Palmar bowing of the flexor retinaculum;
- And hypervascularisation (10).

In a previous study we found that a wrist circumference-dependent cut-off value of the CSA could lead to increased diagnostic accuracy (11). In this study we used the increase of the CSA of the median nerve at wrist level as parameter and evaluated which of three ultrasonographic cut-off values can confirm the clinical diagnosis of CTS the most accurately. These three parameters include a cut-off value of the maximum CSA based on (1) a fixed cut-off value, (2) a wrist circumference-dependent cut-off value, and (3) an intraneural flow-dependent cut-off value (the presence or absence of increased nerve vascularisation).

In earlier studies (12) we used more rigid clinical criteria for diagnosing CTS. Patients were included if they experienced paraesthesias and/or pain in the median nerve-innervated territory, and two or more of the following clinical signs: (1) nocturnal paraesthesias, (2) aggravation of paraesthesias by driving, holding a book or telephone, and (3) a positive Flick sign. However, in our experience, confirmation of presumed CTS is often required by the clinician for patients who do not fulfil all these criteria. In this study we analysed data from three large Dutch teaching hospitals with less strict, but in daily practice more commonly used, inclusion criteria for CTS, as mentioned in paragraph Study Population and Sonography Assessment. Compared to earlier studies, waking up at night due to the symptoms was not mandatory in this study and we did not specify the different types of aggravating activities.

The primary goal of this study was to investigate in how many patients clinical CTS conformation could be achieved by using these three cut-off values and compare performance of these cut-off values. The clinical diagnosis, as defined in the inclusion criteria in the next section, was used as the gold standard.

## Materials and Methods

### Methods

Patients with a clinical suspicion of CTS were referred by their general practitioners between 2018 and 2020. Neurologists in one Dutch university hospital (Radboud university medical centre, Nijmegen) and two Dutch teaching hospitals [Zuyderland hospital, Heerlen and Canisius-Wilhelmina hospital (CWZ), Nijmegen] took the history and included patients if they met the criteria as mentioned in the next paragraph. Ultrasonography was performed and the cross-sectional area (CSA) for the median nerve was measured in all hospitals. The circumference at the distal wrist crease [affected side(s)], height, weight, age, gender and the duration of symptoms were documented. Ultrasonography studies were performed on the same day for each patient and by experienced electrodiagnostic technicians.

The study was approved by the local ethics committee.

#### Study Population and Sonography Assessment

Patients were included if they met all of the following inclusion criteria:

- Over 18 years old;
- Paraesthesia (possibly accompanied by hypaesthesia and/or pain) in the territory of the median nerve;
- Aggravation of complaints by certain activities or wrist movements;
- Reasonable exclusion of other causes based on history-taking and examination.

In patients with bilateral complaints only one side was randomly included. We excluded patients with clinical signs of polyneuropathy, previous surgery or trauma to the wrist and bifid median nerves. Also, pregnant patients and patients with a history of rheumatoid arthritis, diabetes mellitus, hereditary neuropathy with liability to pressure palsies, thyroid disease or alcoholism were excluded.

US studies were performed by neurophysiology technicians with at least 5 years of nerve ultrasound experience. The studies were performed with a Hitachi Aloka Arietta 850 ultrasound system in the Canisius-Wilhelmina hospital (5–17 MHz linear array transducer) and in the Zuyderland hospital (5–18 MHz linear array transducer). In the Radboud university medical centre (Radboudumc) a Fujifilm Sonosite Xporte (5–16 MHz linear array transducer) was used. The main settings of the US machine were: frequency 17–18 MHz, acoustic power 100%, deepness 1.5 cm and focus position 2 cm. Patients were examined in a sitting position with their forearm in supination resting on an examination couch. The median nerve ultrasonography was performed in longitudinal and transverse planes. The inner margin of the hyperechoic rim was outlined by the technicians, as was learned in specialised ultrasound training. The CSA was calculated by the software of the ultrasound system, rounding all measurements to the nearest 0.01 cm^2^. Colour Doppler sonography with no extra manual compression was used to depict potential intraneural blood vessels. The power Doppler box was placed over the nerve with the focus point adjusted to the nerve depth. The colour gain was set to the maximum level for higher sensitivity to flow signals. Intraneural flow was defined as pulsatile focal colour flow signals. Ultrasonographic protocol in the Radboudumc included measurements of both distal and proximal carpal tunnel CSA; we included the largest measured CSA only.

A fixed cut-off value of >11 mm^2^ for the CSA of the median nerve at the wrist level was compared with wrist circumference-dependent cut-off values and cut-off values based on increased nerve vascularisation in the median nerve. In the Dutch CTS guideline (6) a fixed cut-off of 11 mm^2^ for the median nerve CSA at wrist level is mentioned based on a study from Visser et al. (13). The wrist circumference-dependent cut-off value was calculated by an equation, y = 0.88 ^*^ x−4.0, where y is the upper limit of normal of the CSA and x = wrist circumference in centimetres, as described in a previous study (11). If intraneural flow was present a cut-off value of > 12.4 mm^2^ was used, and if absent, the cut-off value was >11.2 mm^2^. These cut-off values were based on the CSA upper limit of normal 95th percentiles in a healthy population (14).

For readability purposes we abbreviated the cut-off values in the rest of this paper as FC for the fixed cut-off value, WDC for the wrist circumference dependent cut-off value and IFC for the intraneural flow related cut-off value.

### Statistics

Statistical analyses were performed using SPSS Statistics Version 26.0. The type of distribution of the data was checked by performing visual analysis of the histograms, the Kolmogorov–Smirnov test and Q-Q plots. Group comparisons for patient characteristics were performed by Chi–Square test (nominal unpaired data), one-way ANOVA analysis for numerical, normally distributed, unpaired data and for non-parametric data the Mann–Whitney *U* (2 groups) or Kruskal–Wallis (>2 groups) test. The categorical data of the three hospitals combined was analysed using McNemar's test for paired data. *p* < 0.05 was considered to be statistically significant.

## Results

Table 1 shows the patient characteristics. A total number of 175 patients were included. There were no statistically significant differences between the hospitals in gender, age, side of included wrist, height, weight, BMI, or the duration of symptoms. Particularly, wrist circumference did not differ between these patient groups. The CSA of the median nerve at wrist was significantly smaller in the Zuyderland hospital compared with CWZ (*p* < 0.001) and compared with the Radboudumc (*p* = 0.042). This value did not differ between CWZ and the Radboudumc (*p* = 0.318).

**Table 1:** Patient characteristics.

	**Zuyderland**	**Radboudumc**	**Canisius-Wilhelmina hospital (CWZ)**	**Total**	***p***
Participants (*n*)	71	41	63	175	
Men/women	22 (31.0%)/49 (69.0%)	9 (22.0%)/32 (78.0%)	22 (34.9%)/41 (65.1%)	53 (30.3%)/122 (69.7%)	0.367^a^
Mean age (y, SD)	53.8 (15.3)	57.0 (12.8)	57.3 (16.3)	55.8 (15.2)	0.347^b^
Left/right	35/36	16/25	32/31	83/92	0.461^a^
Median height (cm, IQ range)	168.0 (12.0)	168.0 (9.0)	169.0 (9.0)	168.6 (10.0)	0.962^c^
Median weight (kg, IQ range)	77.5 (22.0)	78.0 (21.0)	78.0 (20.0)	78.0 (21.0)	0.559^c^
Median BMI (kg/m^2^, IQ range)	27.1 (5.7)	27.1 (5.5)	27.7 (6.2)	27.4 (6.0)	0.210^c^
Median duration symptoms (months, IQ range)	6.0 (20.5)	6.0 (21.8)	12.0 (21.0)	6.0 (22.0)	0.379^c^
Median wrist circumference (cm, IQ range)	17.0 (2.0)	17.0 (2.0)	16.9 (1.7)	17.0 (1.8)	0.950^c^
Minimum-maximum range wrist circumference in cm	14.0-20.0	14.0-19.5	14.4-20.0	14.0-20.0	
Median left wrist circumference (cm, IQ range)	17.0 (1.2)	17.8 (1.4)	16.8 (1.4)		
Mean right wrist circumference (cm, SD)	17.0 (2.7)	17.0 (1.8)	17.2 (1.8)		
Median CSA wrist (mm^2^, IQ range)	10.3 (2.7)	12.3 (4.8)	12.2 (5.4)	11.1 (4.3)	<0.001^c†^
Median CSA wrist left (mm^2^, IQ range)	10.3 (2.3)	12.8 (6.5)	12.4 (4.3)		
Median CSA wrist right (mm^2^, IQ range)	9.8 (2.6)	12.0 (4.2)	12.0 (6.0)		
Intraneural flow	6 (8.5%)	0	13 (20.6%)	19 (10.9%)	0.001^d^
Intraneural flow left	1 (1.4%)	0	7 (11.1%)	8 (4.6%)	
Intraneural flow right	5 (7.0%)	0	6 (9.5%)	11 (6.3%)	
Intraneural flow absent	65 (91.5%)	41 (100%)	50 (79.4%%)	156 (89.1%)	

a *Pearson Chi-Square test*.

b *One-way between groups ANOVA analysis*.

c *Kruskal-Wallis test*.

d *Likelihood ratio Chi-Square test*.

† *In the Zuyderland hospital compared to CWZ, and the Zuyderland hospital compared to the Radboudumc, a statistically significant different CSA at the wrist, p < 0.001 and p = 0.042, respectively, was found. The CSA at the wrist in the Radboudumc compared to CWZ was not statistically significantly different (p = 0.318) (Mann–Whitney U-test)*.

### Patient Data

In order to assess the primary goals of this study, data of the three hospitals was combined. We compared the total number of abnormal ultrasonography results using the three different parameters as discussed. In Table 2 the results are presented.

**Table 2:** Combined results of ultrasonography of the three hospitals for the fixed cut-off, the wrist circumference-dependent cut-off (WDC) and the cut-off based on intraneural flow (IFC).

**Sonography**		**Total number of wrists (*n* = 175)**	**Zuyderland (*n* = 71)**	**Radboudumc (*n* = 41)**	**CWZ (*n* = 63)**
Abnormal	FC	88 (50.3%)	21 (29.6%)	23 (56.1%)	44 (69.8%)
	WDC	97 (55.4%)	29 (40.8%)	25 (61.0%)	43 (68.3%)
	IFC	82 (46.9%)	20 (28.2%)	23 (56.1%)	39 (61.9%)

**Figure 1:**
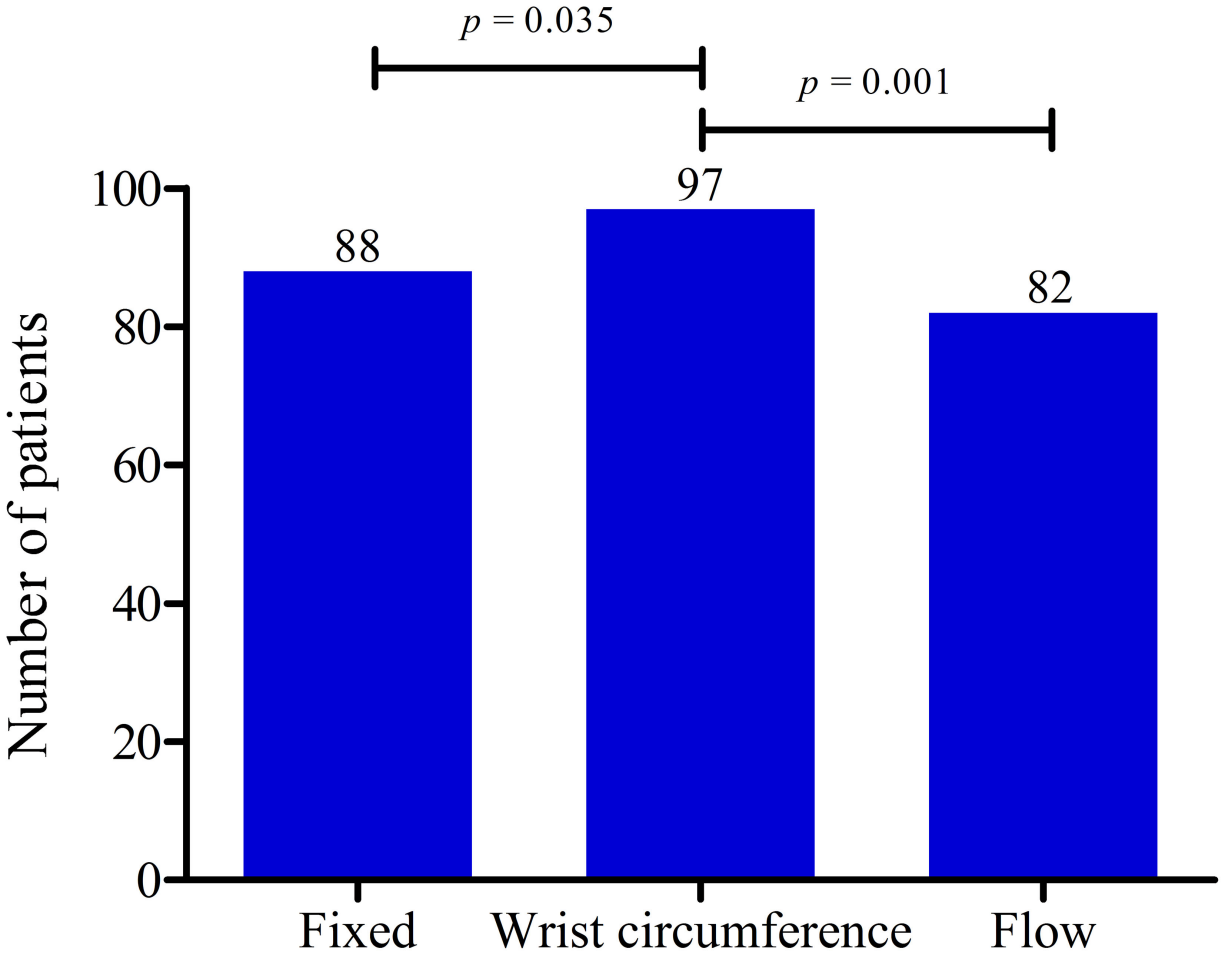
Data of three hospitals combined showing the number of patients with abnormal ultrasonography (US) results. Comparison of the fixed cut-off value (FC), the wrist circumference-dependent cut-off value (WDC) and the flow-dependent cut-off value (IFC). US results were more often abnormal both with the FC (*p* = 0.031) and the WDC (*p* = 0.001) compared with the IFC. *P*-values calculated with McNemar's test.

As shown in Figure 1, ultrasonography was significantly more often abnormal while using the WDC compared with the FC (*p* = 0.035) or the IFC (*p* = 0.001).

The following paragraphs give insight in the hospital-specific data.

#### Zuyderland Hospital

We collected data from 75 patients. Ten bifid median nerves in nine patients led to four exclusions. We eventually included 71 patients. Table 3 shows the data of the performed tests of the Zuyderland hospital. Interestingly, ultrasonography was abnormal in only 28.2% of the examined wrists when using the IFC, while 40.8% were considered abnormal when the WDC was used.

**Table 3:** Results of ultrasonography in the Zuyderland hospital for the fixed cut-off (FC), the wrist circumference-dependent cut-off (WDC) and the cut-off based on intraneural flow (IFC).

	**FC**	**WDC**	**IFC**
US abnormal	21 (29.6%)	29 (40.8%)	20 (28.2%)
US abnormal left	12	16	12
US abnormal right	9	13	8

#### Radboud University Medical Centre

Data from 48 patients was collected. We excluded one patient because of missing data points, two patients with bifid median nerves, two posttraumatic CTS patients and two patients because of prior wrist surgery. Forty-one patients were included. Table 4 shows the hospital-specific results. 56.1–61.0% of the 41 examined wrists showed abnormal US results.

**Table 4:** Results of ultrasonography in the Radboudumc for the fixed cut-off (FC), the wrist circumference-dependent cut-off (WDC) and the cut-off based on intraneural flow (IFC).

	**FC**	**WDC**	**IFC**
US abnormal	23 (56.1%)	25 (61.0%)	23 (56.1%)
US abnormal left	9	9	9
US abnormal right	14	16	14

#### Canisius-Wilhelmina Hospital (CWZ)

Data of 72 patients was obtained. Seven patients were excluded because of bifid median nerves, Two patients had a medical history of wrist arthrosis. Sixty-three patients were included. In Table 5 the results of CWZ are presented. Ultrasound test results were abnormal in 61.9–69.8%.

**Table 5:** Results of ultrasonography in the Canisius-Wilhelmina hospital for the fixed cut-off (FC), the wrist circumference-dependent cut-off (WDC) and the cut-off based on intraneural flow (IFC).

	**FC**	**WDC**	**IFC**
US abnormal	44 (69.8%)	43 (68.3%)	39 (61.9%)
US abnormal left	24	22	22
US abnormal right	20	21	17

### Comparison of the Ultrasonography Parameters

The WDC considered 12 median nerves to be abnormal while the FC did not, and 18 while the IFC did not. The wrist circumference of these 12 and 18 wrists turned out to be significantly smaller (*p* < 0.001) when compared with the rest of the wrist circumferences as shown in Table 6. The mean CSA between these groups was not significantly different. Most of these patients (9/12 in the normal fixed group and 10/18 in the normal flow group) were examined at the Zuyderland hospital.

**Table 6:** Patient characteristics of 12 and 18 patients with an abnormal ultrasonography (US) result while using the wrist circumference dependent cut-off value but normal US results using the fixed and the flow-dependent cut-off value, respectively.

	**Normal fixed**	**Others**	***p***	**Normal flow**	**Others**	***p***
Wrists (*n*)	12	163		18	157	
Men/Women	2/10	51/112	0.287^a^	3/15	50/107	0.184^a^
Mean age (years, SD)	54.5 (12.0)	55.9 (15.4)	0.760^b^	52.8 (12.9)	56.1 (15.4)	0.374^b^
Left/right	5/7	78/85	0.679^a^	7/11	76/81	0.444^a^
Median height (cm, IQ range)	163.0 (11.8)	168.0 (10.3)	0.268^c^	164.0 (11.0)	169.0 (10.8)	0.061^c^
Median weight (kg, IQ range)	69.5 (15.3)	78.5 (20.0)	0.013^c^	72.0 (16.3)	78.5 (20.3)	0.071^c^
Median BMI (kg/m^2^, IQ range)	25.4 (4.1)	27.6 (6.0)	0.030^c^	26.8 (4.9)	27.9 (5.9)	0.267^c^
Median duration symptoms (months, IQ range)	4.0 (7.0)	6.0 (22.0)	0.078^c^	4.0 (10.0)	6.0 (22.0)	0.162^c^
Median wrist circumference (cm, IQ range)	16.0 (0.7)	17.0 (1.6)	<0.001^c^	16.0 (0.7)	17.0 (1.6)	<0.001^c^
Minimum-maximum range wrist circumference in cm	14.0–16.7	14.0–20.0		14.4–18.0	14.0–22.0	
Median CSA (mm^2^, IQ range)	10.3 (0.6)	11.7 (4.4)	0.281^c^	11.0 (1.7)	11.3 (4.8)	0.618^c^

a *Pearson Chi-Square test*.

b *Unpaired t-test*.

c *Mann–Whitney U-Test*.

## Discussion

In this cross-sectional study a wrist circumference-dependent cut-off value for the upper limit of normal of the CSA at the wrist led to more abnormal US results than either a fixed upper limit of normal of 11 mm^2^ (*p* = 0.035) or a cut-off value based on intraneural flow (*p* = 0.001) did.

As shown in an earlier study, a WDC of the CSA may augment diagnostic accuracy of ultrasonography in CTS patients (11). We found that this parameter considered more US to be abnormal than a FC or an IFC did. The mean CSA of the median nerves of the evaluated patients was relatively low in this study. In the Zuyderland hospital the mean CSA was significantly lower than in the Canisius-Wilhelmina hospital (*p* < 0.001) and the Radboudumc (*p* = 0.042). This lower mean CSA in the Zuyderland hospital leads to a very low number of abnormal US in the Zuyderland hospital, ranging from 28.2 to 40.8%. Looking at the small subgroup of patients with abnormal US results while using the WCD and normal US results while using the other two cut-off methods, a statistically significantly smaller wrist circumference was noticed, as is shown in Table 6. These results may point out that a cut-off value based on the wrist-circumference adds the most value in people with a smaller wrist circumference when compared with the other analysed US parameters in this study.

The lack of a gold standard is an important problem in the diagnosis of CTS and this complicates research regarding CTS (15). In literature, clinical signs and symptoms, NCS and (surgical) outcome are used as reference standards (15). CTS is a clinical diagnosis and without signs and symptoms an individual cannot be diagnosed with CTS but can have abnormal US/NCS outcomes. False positives as well as false negatives are therefore present in groups of patients with clinically defined CTS but also in groups of patients with e.g., abnormal US and/or abnormal NCS outcomes. A positive effect of surgical treatment is another possible reference standard. However, even sham operations could have a positive (placebo) effect. Furthermore, in a previous study we showed that patients with clinically defined CTS and normal NCS noted a significant reduction of complaints after carpal tunnel release (16). For the examined wrists in this study the same problem of false positives and false negatives exists and the less strict inclusion criteria are probably the cause of the low sensitivity of US in this study.

Several studies suggest that increased nerve vascularisation in the median nerve, as evaluated by colour Doppler sonography, is associated with (severity of) carpal tunnel syndrome (10). However, one study showed an increased median nerve vascularisation prevalence of 36% in 60 healthy individuals (14). Surprisingly, in this study, median nerve vascularisation was only present in 10.9% of the participants and in the Radboudumc absent in all participants. This is not in line with literature where increased nerve vascularisation is reported in 41–95% of CTS patients (10, 17). This may be explained by differences in techniques while performing US (e.g., manual compression) or differences in settings and/or Doppler sensitivity between US devices. A cut-off value based on nerve median nerve vascularisation seemed to be the least favourable cut-off compared with the other investigated parameters in this study.

There are several limitations to this study. Firstly, it is important to bear in mind the possible bias in our data caused by our more liberal inclusion criteria for investigating carpal tunnel syndrome. We did not mean to change the clinical criteria for CTS in any way, but our goal was to investigate a patient population, as may be encountered in daily outpatient clinical practice, as described in the introduction. Secondly, the neurophysiology technicians performing the US were not blinded. The technicians may have expected to find enlargement of the median nerve, however, compared to an earlier Dutch study the median CSA of the median nerve at wrist level seems to be lower in this study, especially in the Zuyderland hospital (13). Concerning ultrasonography, we did not measure intra- or interobserver variability of the measurements. Earlier studies reported good agreement of CSA measurements of the median nerve (18, 19) but we cannot fully exclude interobserver variability due to variation in outlining the nerve contour (20), particularly because of the significantly smaller CSA found in the Zuyderland hospital. Only in the Radboudumc the distal carpal tunnel was visualised, in the other hospitals the median nerve was visualised only in the proximal and middle part of the carpal tunnel. However, in this study the percentage of abnormal US in the Canisius-Wilhelmina hospital and the Radboudumc were comparable. Furthermore, we cannot exclude slight differences in interhospital interpretations concerning the inclusion criteria.

In conclusion, a WDC for the CSA of the median nerve at the wrist appears to have a higher sensitivity than a FC or IFC in CTS patients in clinical practice, who do not always fulfil more rigid clinical criteria for the clinical diagnosis CTS. A cut-off for the CSA of the median nerve based on the wrist-circumference may at present be the most powerful approach in patients with a smaller wrist circumference.

## Data Availability Statement

The raw data supporting the conclusions of this article will be made available by the authors, without undue reservation.

## Ethics Statement

The studies involving human participants were reviewed and approved by Commissie Mensgebonden Onderzoek regio Arnhem-Nijmegen. The ethics committee waived the requirement of written informed consent for participation.

## Author Contributions

TO, FD, JM, and WV: conception and design of study. TO, RBe, and JW: acquisition of data. TO, FD, RBe, JW, RBa, JM, and WV: analysis and/or interpretation of data. TO: drafting of manuscript. TO, FD, RBe, JW, RBa, JM, and WV: revising manuscript. All authors read and approved the final version.

## Conflict of Interest

The authors declare that the research was conducted in the absence of any commercial or financial relationships that could be construed as a potential conflict of interest.

## Abbreviations

CTS: carpal tunnel syndrome
CSA: cross-sectional area
FC: fixed cut-off value
IFC: intraneural flow related cut-off value
IQ: interquartile range
NCS: nerve conduction studies
ULN: upper limit of normal
US: ultrasonography
WDC: wrist circumference-dependent cut-off value.
